# Metabolic Dysregulation in Laminopathies: Implications for Heart Failure and Cardiac Health

**DOI:** 10.1007/s11897-025-00735-8

**Published:** 2026-02-02

**Authors:** T. Torfs, R. J. A. Veltrop, G. Lezzoche, S. R. B. Heymans, J. A. J. Verdonschot, M. Nabben

**Affiliations:** 1 Department of Cardiology, CARIM Cardiovascular Research Institute Maastricht, Maastricht University, Maastricht, the Netherlands; 2 Department of Biochemistry, CARIM, Maastricht University, Maastricht, The Netherlands; 3 Department of Cardiology, Division Heart and Lungs, University Medical Center Utrecht, Utrecht, the Netherlands; 4 Regenerative Medicine Utrecht, University Medical Center Utrecht, Utrecht, The Netherlands; 5 Netherlands Heart Institute (NLHI), Utrecht, The Netherlands; 6 LMNAcardiac.org, LMNA Patient Organization, Maastricht, The Netherlands; 7 European Reference Network for Rare, Low Prevalence and Complex Diseases of the Heart (ERN GUARD-Heart), Soest, The Netherlands; 8 Centre of Cardiovascular Research, Centre for Molecular and Vascular Biology, University of Leuven, Leuven, Belgium; 9 Department of Clinical Genetics, Maastricht University Medical Center+, Maastricht, the Netherlands; 10 Maastricht University, Universiteitssingel 50, Maastricht, 6229 ER The Netherlands

## Abstract

**Purpose of the Review:**

Laminopathies are a diverse group of genetic disorders caused by variants in nuclear lamina proteins, with heart failure being a major cause of morbidity and mortality in patients. By shifting the perspective from distinct clinical phenotypes to a continuous spectrum of metabolic observations in laminopathies, this review aims to elucidate the molecular mechanisms underlying metabolic dysregulation in laminopathies and their impact on cardiac health.

**Recent Findings:**

Emerging evidence links A-type lamins to cellular metabolism, with systemic metabolic changes such as lipodystrophy, insulin resistance and hypertriglyceridemia often detected in laminopathy patients before cardiac symptoms onset. At the molecular level, *LMNA *variants disrupt the regulation of genes involved in fatty acid, oxidation glucose utilization, and mitochondrial function. These disturbances increase cardiomyocyte susceptibility, promoting fibrosis and apoptosis.

**Summary:**

Metabolic dysregulation is a recurring observation across the laminopathy spectrum. Targeting metabolic pathways shows preclinical promise for improving cardiac outcomes in patients.

## Introduction

 Laminopathies are a heterogeneous group of disorders caused by variants in nuclear lamina proteins, characterized by marked clinical variability and multisystem involvement, including the heart. Although lamins are primarily known for their structural role in maintaining nuclear architecture, growing evidence links them to metabolic regulation and cardiac function (Table 1) [45–47]. The latter is particularly relevant as cardiac involvement is a major cause of morbidity and mortality in laminopathy patients [48].

**Table 1:** Metabolic observations in different laminopathy phenotypes

		Lipodystrophy	Insulin resistance	Hypertriglyceridemia	Glucose intolerance	Muscle hypertrophy	Myagia/weakness	Premature aging	Cardiac involvement
Striated muscle diseases	EDMD	- [1]	X [2]	X [3–5]	ND	X	X [1]	- [1]	X [6, 7]
	DCM	- [8]	ND	X [9]	ND	- [10]	- [10]	- [10]	X [6, 7]
	LGMD1B	- [8]	- [3]	ND	- [3]	- [3]	X [3]	- [3]	X [6]
Adipose tissue disorders	FPLD	X [11]	X [12]	X [12, 13]	X [3, 14]	X [15]	- [16]	X [17]	X
	MAD	X [18]	X [19, 20]	X [21]	X [19, 20]	ND	X [22]	X [23]	X [24]
	*LMNA*-related metabolic syndrome	X [21, 25]	X [26]	X [26]	X [26, 27]	- [26]	X [27]	- [26]	X [27, 28]
Progeroid syndromes	HGPS	X [25]	X [29]	X [30, 31]	X [32, 33]	- [34]	X [34]	X [31, 32]	X [24]
	AWS	- [35, 36]	X [37]	X [37, 38]	X [32, 33]	ND	X [39]	X [39]	X [40, 41]
Nerve and skin disorders	CMT2	- [42]	ND	ND	ND	ND	X [43]	ND	X [44]

The cross table presents an overview of the various metabolic and systemic observations associated with the different laminopathy phenotypes. The diversity in clinical presentations underlines the spectrum nature of laminopathies. (X) indicates the presence of a particular (metabolic) observation. (-) indicates the absence of the metabolic observation. (ND) indicates no reported data. Abbreviations: EDMD: Emery-Dreifuss Muscular Dystrophy; DCM: Dilated Cardiomyopathy; LGMD1B: Limb-Girdle Muscular Dystrophy 1B; FPLD: Dunnigan-type Familial Partial Lipodystrophy; MAD: Mandibuloacral Dysplasia; HGPS: Hutchinson-Gilford Progeria Syndrome; AWS: Atypical Werner’s Syndrome; CMT2: Charcot-Marie-Tooth disease Type 2

Disturbances in systemic metabolism, especially in adipose tissue and skeletal muscle, can have a major impact on the heart. In adipose tissue, impaired lipid storage, altered adipokine release, and chronic inflammation create a circulating lipotoxic and inflammatory environment that affects cardiac metabolism and structure. Skeletal muscle abnormalities, including excess lipid accumulation, can contribute to insulin resistance and metabolic stress [49, 50]. These processes promote cardiomyocyte injury, mitochondrial dysfunction, and fibrosis, linking peripheral metabolic dysfunction to cardiac pathology [51]. In laminopathies, such systemic disturbances, combined with direct nuclear defects from *LMNA* variants, may accelerate the development of cardiomyopathy [52].

Currently, up to 18 laminopathy phenotypes are recognized and grouped into five major classes based on affected tissues and clinical presentation: (1) striated muscle diseases (e.g., Emery-Dreifuss muscular dystrophy, dilated cardiomyopathy, limb-girdle muscular dystrophy 1B); (2) adipose tissue disorders (e.g., Dunnigan-type familial partial lipodystrophy, mandibuloacral dysplasia); (3) premature aging syndromes (e.g., Hutchinson-Gilford progeria syndrome, atypical Werner’s syndrome, atypical progeroid syndrome); (4) nerve and skin disorders (e.g., Charcot-Marie-Tooth type 2 peripheral neuropathy); and (5) overlapping phenotypes involving multiple tissues [52–55].

The nuclear lamina primarily consists of A-type lamins (lamin A and C, encoded by *LMNA*) and B-type lamins (lamin B1 and B2, encoded by *LMNB1* and *LMNB2*). Although both share structural functions, they differ in sequence, expression patterns, and biological roles. B-type lamins are ubiquitously expressed and essential for cell survival, whereas A-type lamins exhibit tissue-specific expression and are predominantly found in differentiated cells [56, 57].

Beyond their structural role, A-type lamins regulate lipid metabolism and adipocyte differentiation through interactions with transcriptional regulators. *LMNA* variants or abnormal lamin processing disrupt these interactions, leading to metabolic disturbances such as lipodystrophy, insulin resistance, and altered glucose and fatty acid metabolism (Table 1). These abnormalities often precede cardiac symptoms in laminopathies [21, 58, 59] and may contribute to cardiac dysfunction by impairing energy supply and increasing cardiomyocyte vulnerability to stress [60]. In contrast, B-type lamin deficiencies primarily cause nuclear fragility and cell death rather than defined metabolic syndromes [61, 62]. This review therefore only discusses A-type lamins.

Clinically, heart failure in laminopathy patients is more aggressive and less responsive to standard therapies compared to other genetic dilated cardiomyopathy genotypes, underscoring the need for early and regular cardiac monitoring. Progression to advanced heart failure can be rapid and malignant, with a high risk of sudden cardiac death even in cases of mild ventricular dysfunction [63, 64].

Furthermore, genetic screening of patients with metabolic syndrome has revealed a 3% prevalence of *LMNA *variants, suggesting a potential role in disease predisposition [65, 66]. Insulin resistance and dyslipidemia, common in laminopathies, also overlap with metabolic syndrome features [27]. These observations highlight the importance of elucidating the molecular mechanisms linking lamin dysfunction, metabolic disturbances, and cardiac health.

## Cardiac Metabolism and Energy Supply in Laminopathies

Under normal physiological conditions, the healthy heart demonstrates a remarkable metabolic flexibility, with fatty acids metabolism contributing to 60–70% of the total ATP production [67, 68]. Alongside fatty acids, glucose is another main contributor to the heart’s energy supply and can be upregulated in response to insulin or during ischemic conditions [69, 70]. Glucose is obtained from the blood stream and metabolised through glycolysis to produce pyruvate which enters the tricarboxylic acid (TCA) cycle to generate ATP [71].

The heart can also utilise alternative substrates such as lactate, generated from anaerobic glycolysis as well as ketone bodies and amino acids, particularly during conditions of increased work load or metabolic stress [72–74]. Metabolic stress refers to a state in which the energy demand exceeds the energy supply, leading to the accumulation of metabolites such as lactate, phosphate, and hydrogen ions in cardiomyocytes [75, 76]. Metabolic flexibility is regulated by factors such as hormonal signals, substrate availability, oxygen availability and the energy state of the cell. Metabolic flexibility is essential for adapting to varying physiological demands, such as exercise and fasting [77].

**Fig. 1:**
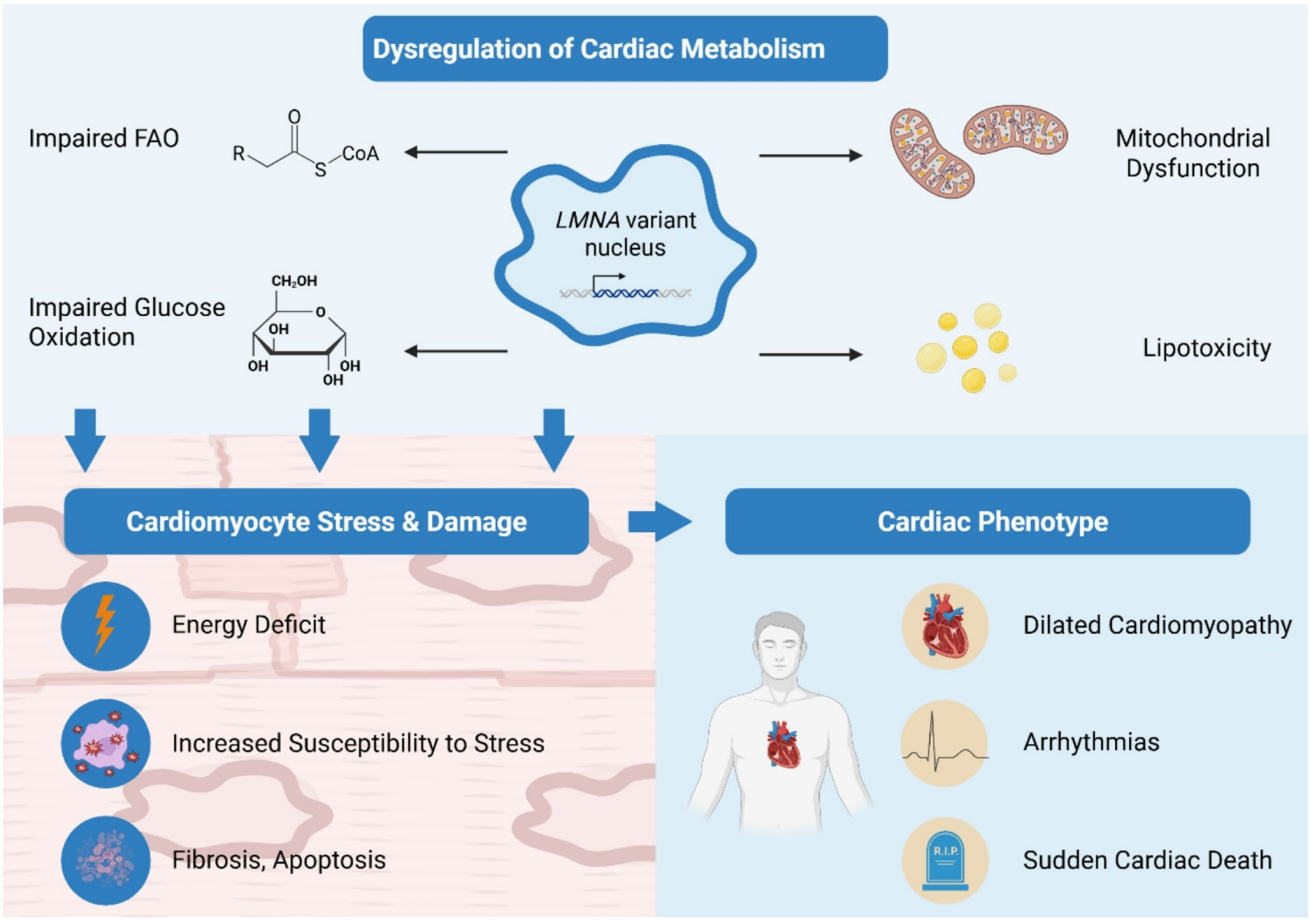
Impact of Laminopathies on Cardiac Health. Genetic variants in the *LMNA* gene disrupt nuclear architecture and gene expression, leading to dysregulation of cardiac metabolism (impaired fatty acid oxidation, glucose utilisation, and mitochondrial dysfunction). These metabolic disturbances increase cardiomyocyte susceptibility to stress, promoting fibrosis, apoptosis and ultimately cardiac dysfunction. Systematic abnormalities such as lipodystrophy, insulin resistance, and hypertriglyceridemia, further exacerbate cardiac risk. Abbreviations: FAO: fatty acid oxidation. Image created in Biorender.com

Cardiac involvement, particularly heart failure, is a major concern in laminopathies (Fig. 1). The most prominent manifestations are in DCM, EDMD, and LGMD1B, all of which exhibit a high incidence of heart failure and arrhythmias [48, 78].

The molecular mechanisms underlying heart failure in *LMNA*-related DCM are not fully understood but may involve altered gene expression, impaired mechanotransduction, nuclear envelope dysfunction, fibrosis, and cardiomyocyte apoptosis [79]. The ‘altered gene expression’ hypothesis suggests that *LMNA* variants disrupt the regulation of numerous genes and signalling pathways, including TGF-β, AKT-mTORC1, and PDGF pathways, developmental regulators (e.g. EMT-related genes and BMP signalling components), genes associated with non-cardiac lineages and nuclear functions, X-linked genes (e.g., *GPC3*), imprinted genes (e.g., *SNRPN*, *MEG3*), long non-coding RNAs (e.g., *XIST*), and DNA damage response genes [80, 81].

Beyond these transcriptional disruptions, *LMNA* variants also affect the expression of genes regulating key metabolic processes, such as fatty acid oxidation, glucose utilisation, and mitochondrial energy production, causing early metabolic inflexibility and energy deficits in cardiomyocytes [82–84]. These metabolic disturbances can increase the susceptibility of cardiomyocytes to stress and damage, thereby contributing to the development and progression of heart failure and arrhythmias. As cardiac dysfunction advances, further metabolic changes can occur, creating a vicious cycle where metabolic and functional abnormalities reinforce each other [69, 85].

While cardiomyocytes are early involved in the disease pathogenesis, altered gene expression also occurs in cardiac fibroblasts, endothelial cells and immune cells, with changes in cell-cell communication mediated through extracellular matrix modifications [86]. For instance, fibrosis results from activation of TGF-$\:\beta\:$ signalling and extracellular matrix remodelling, partly driven by *LMNA* deficiency in cardiac fibroblasts in *Lmna* mouse models [79, 87]. Endothelial cells show increased stress and mesenchymal markers, suggesting endothelial-to-mesenchymal transition, contributing to cardiac fibrosis [80, 88]. This widespread altered expression of genes contributes to dysregulated cellular functions and differentiation patterns, leading to cardiomyocyte death, fibrosis, and electrical remodelling, which are hallmarks of *LMNA*-related DCM, though the precise mechanisms continue to be investigated.

## Lipid Metabolism Dysregulation in Laminopathies

### Early Metabolic Changes in Lipodystrophies and Muscular Laminopathies

**Fig. 2:**
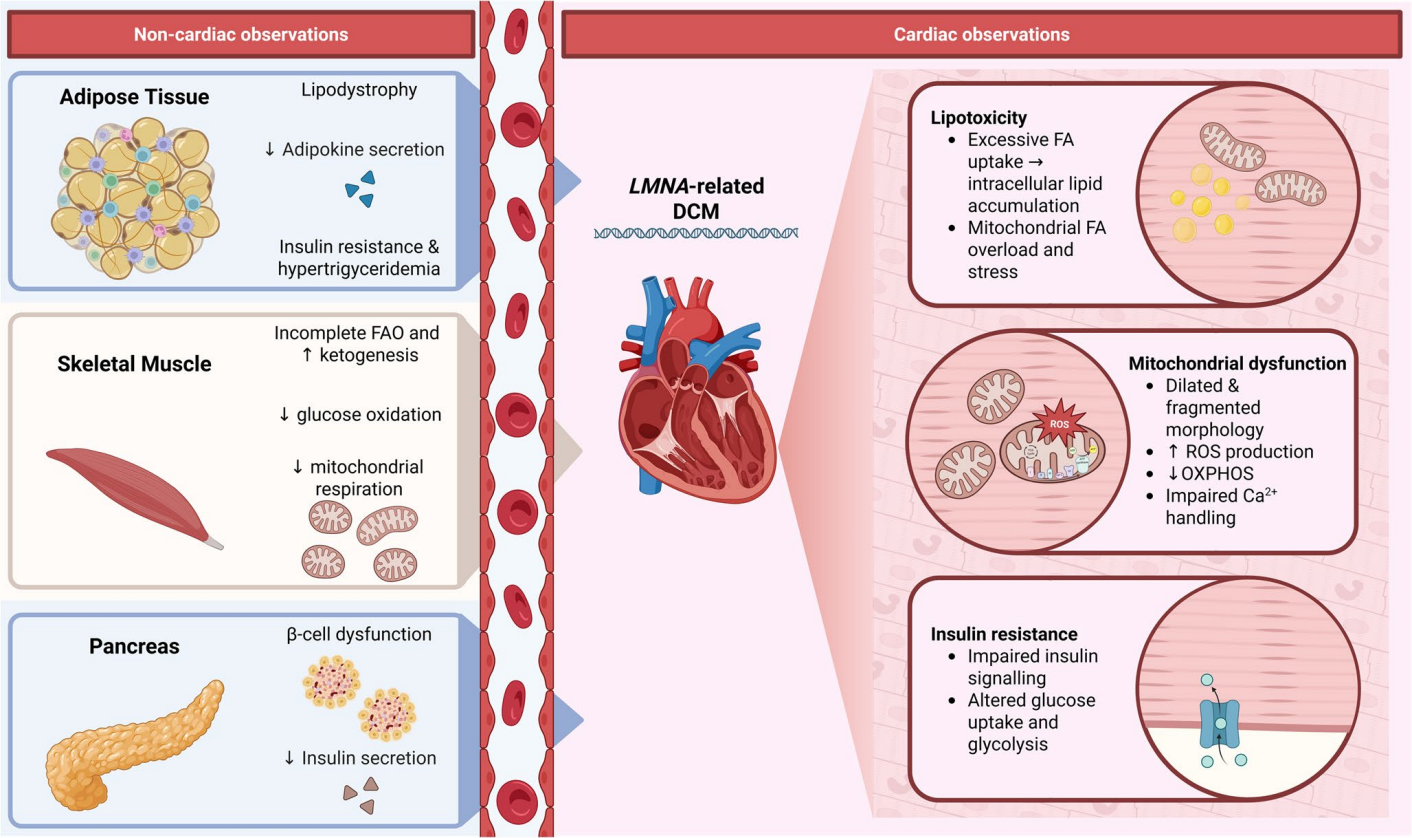
Pathways from *LMNA *Variants to Cardiac Dysfunction via Systemic and Cardiac Metabolic Dysregulation. This schematic summarises the effects of *LMNA *variants leading to dilated cardiomyopathy (DCM) by integrating systemic (non-cardiac) and cardiac metabolic dysfunction. *LMNA *variants drive lipodystrophy, reduced adipokine secretion, and insulin resistance in adipose tissue; β-cell dysfunction and reduced insulin secretion in the pancreas; and incomplete fatty acid oxidation (FAO), impaired glucose oxidation, and mitochondrial dysfunction in skeletal muscle. These systemic alterations contribute to myocardial metabolic derangements, including excessive fatty acid (FA) uptake, intracellular lipid accumulation, and mitochondrial FA overload (lipotoxicity). Cardiac-specific consequences include mitochondrial dysfunction, characterised by altered morphology, increased reactive oxygen species (ROS) production, compromised oxidative phosphorylation (OXPHOS), and disturbed Ca ^2+^ handling. Additionally, impaired insulin signalling, altered glucose uptake and glycolysis further exacerbate cardiomyocyte metabolism. Together, these interconnected metabolic changes provide a mechanistic basis for *LMNA*-related DCM pathogenesis. Figure created in Biorender.com

Lipid metabolism involves the acquisition, synthesis and degradation of lipids within cells, and plays a crucial role in energy storage, membrane structure and cell signalling [89]. In laminopathies, dysregulation of lipid metabolism is most evident in adipose tissue disorders such as FPLD and MAD, but has also been reported in muscular laminopathies (DCM, EDMD, LGMD1B) and progeroid syndromes like HGPS. No direct reports addressing lipid metabolism in CMT2 were available (see Table 1; Fig. 2).

FPLD and MAD share considerable clinical and metabolic overlap, as both are *LMNA*-related adipose tissue disorders characterised by lipodystrophy, insulin resistance, hypertriglyceridemia, and increased risk of cardiovascular complications [12, 17]. Both FPLD and MAD show reduced levels of adipokines such as leptin and adiponectin, which normally exert anti-inflammatory and cardioprotective effects [90, 91]. This decrease may foster a pro-inflammatory environment which exacerbates atherosclerosis and cardiac dysfunction. Cardiac involvement in FPLD and MAD is not limited to premature atherosclerosis but also frequently includes cardiomyopathy [24, 92, 93].

Beyond these adipose tissue defects, disturbances in lipid metabolism are also observed in EDMD and LGMD1B. In these conditions, increased yet incomplete fatty acid oxidation and upregulated ketogenesis have been observed in skeletal muscle [3, 94]. At the molecular level *LMNA* variants affect nuclear architecture and chromatin organisation which specifically impact the regulation of lipid-handling genes [46, 95]. These structural and chromatin changes translate into specific disruptions in lipid regulatory pathways. A-type lamins regulate lipid metabolism by anchoring transcriptional regulators such as sterol regulatory element-binding protein 1 (SREBP1) at the nuclear lamina, thereby controlling genes involved in lipid synthesis and adipocyte differentiation [96, 97]. Disruption of this interaction impairs transcription of adipocyte-related genes, including peroxisome proliferator-activated receptor gamma (PPARγ) [98–102]. Dysregulation of SREBP1 signalling impairs the balance between fatty acid synthesis and oxidation, leading to subsequent lipotoxicity. This results in mitochondrial dysfunction, reducing the capacity for efficient $\:\beta\:$-oxidation and ATP production [96, 103–105]. Accumulated prelamin A can also bind transcription factor Sp1, altering extracellular matrix gene expression essential for adipose lineage differentiation. These changes lead to smaller lipid vesicles, reduced adipocyte differentiation, and a lipodystrophic phenotype [99]. Consequently, *LMNA* variants or abnormal lamin processing result in metabolic disturbances such as lipodystrophy and altered fatty acid metabolism (Table 1). In contrast, B-type lamin deficiencies primarily cause nuclear fragility and cell death rather than defined metabolic syndromes [9, 10]. Together, these defects can result in metabolic disturbances, lipid accumulation, and increased reliance on ketone bodies for energy [101]. The resulting lipotoxicity and cellular stress further exacerbate muscle dysfunction and contribute to the progression of cardiomyopathy and muscle weakness in these laminopathies [3, 4, 106].

HGPS exhibits decreased systemic fat storage and a reduced proportion of adipose tissue relative to lean body mass. Leptin, a peptide hormone synthesised by adipocytes and involved in energy expenditure, was found to be downregulated in HGPS patients [33]. Limited case reports have described hyperlipidaemia, hypertriglyceridemia and hypercholesterolemia in HGPS patients, suggesting impaired lipid clearance or alterations in systemic lipid metabolism [30, 31]. Similarly, hypertriglyceridemia has been documented in case reports of AWS [37, 38].

In *LMNA*-related DCM, fat infiltration has been reported in cardiac muscle, particularly as epicardial and fibrofatty infiltration [107]. *LMNA* variant carriers exhibit fatty degeneration in the right ventricular epicardium; and parts of the cardiac conduction system, including the atrioventricular (AV) node, which may contribute to arrhythmias and contractile dysfunction. This epicardial fat accumulation occurs together with myocardial fibrosis and may play a role in disease progression and mechanical-electrical disruption in *LMNA*-related DCM [108, 109].

### Lipidomic Signatures and Cardiac Risk

Lipidomic studies have identified distinct serum profiles in presymptomatic *LMNA* variant carriers, suggesting that changes in lipid metabolism may precede the development of cardiac dysfunction [9]. The lipidomic profiles were characterised by increased phosphatidylethanolamines (PE) and decreased odd-chain triglycerides, and have been associated with decreased cardiac function as assessed by cardiac MRI [9]. These lipid alterations correlate with reduced contractility and altered myocardial structure [9]. Notably, these observations in lipidomic serum profiles are not exclusive to *LMNA*-related DCM and have been reported in other types of DCM as well, indicating potentially shared lipid metabolic perturbations across different DCM aetiologies [9, 101, 110].

Mechanistically, dysregulation of PE metabolism has been shown to directly affect cardiac contractility and myocardial structure. Studies in Drosophila melanogaster demonstrate that cardiac-specific impairments in PE biosynthesis disrupt membrane homeostasis, causing heart tube constriction and impaired contractile function, highlighting how PE imbalances may alter cardiomyocyte membrane integrity and signalling pathways critical for contraction [111]. In this context, increased circulating or tissue PE levels in *LMNA* variant carriers may reflect compensatory remodeling of membrane phospholipids in response to early nuclear–cytoskeletal stress, potentially altering sarcolemmal fluidity and calcium-handling efficiency. Furthermore, decreased odd-chain triglycerides are associated with mitochondrial dysfunction and clinical cardiomyopathy, suggesting that reduced availability of these lipids may limit anaplerotic substrate supply to the tricarboxylic acid (TCA) cycle, thereby impairing myocardial energy production and structural maintenance [112]. Lipid metabolic imbalances, are well-known to induce lipotoxicity, insulin resistance, and mitochondrial dysfunction in the heart, which contribute to contractile impairment and adverse cardiac remodelling [113]. Together, these findings suggest that altered PE and odd-chain triglyceride metabolism may converge on mitochondrial and membrane-dependent pathways that underlie early contractile dysfunction in *LMNA*-associated cardiomyopathy.

In contrast, a FPLD mouse model study (*Lmna*^*∆K32/+*^) did not show signs of lipid dysregulation in serum triglycerides, cholesterol, or fat disposition [114], underlining the need for further investigation.

Lipid profile disturbances observed across various laminopathies demonstrate the role of A-type lamins in regulating lipid metabolism and energy homeostasis [115]. Distinct patterns of dyslipidaemia, lipoatrophy, insulin resistance, and altered adipokine levels reflect the underlying molecular disturbances caused by nuclear envelope dysfunction [3, 83, 116, 117]. Tissue-specific manifestations, ranging from severe lipodystrophy in FPLD and MAD, to impaired fatty acid oxidation in EDMD and LGMD1B, and the lipid disturbances in progeroid syndromes [20, 116–118], suggest that *LMNA* variants disrupt fatty acid metabolism by altering the expression of genes involved in mitochondrial function, lipid handling, and oxidative capacity [3, 117, 119]. Understanding how nuclear envelope dysfunction leads to distinct metabolic disturbances in different tissues may be essential for developing targeted interventions for laminopathies.

## Glucose Metabolism and insulin Resistance in Laminopathies

### Clinical Evidence and Underlying Mechanisms

In addition to alterations in lipid metabolism, increasing evidence suggests that *LMNA* variants also affect glucose metabolism, contributing to systemic insulin resistance. Insulin resistance is a common manifestation across various laminopathy phenotypes, including EDMD, MAD, *LMNA*-related DCM, HGPS, and AWS, often contributing to systemic metabolic dysfunction, and is particularly pronounced and severe in FPLD and LGMD1B [3, 106]. No direct reports about glucose metabolism in CMT2 were available (see Table 1; Fig. 2).

Peripheral insulin resistance leads to impaired insulin-mediated suppression of adipose tissue lipolysis, resulting in increased circulating free fatty acids. Elevated free fatty acids promote lipotoxicity in cardiomyocytes by driving excessive fatty acid uptake and oxidation, which may overload mitochondrial capacity and generate ROS. This lipotoxic stress directly contributes to mitochondrial dysfunction and causes energy deficits in the heart [120, 121]. Additionally, insulin resistance disrupts cardiac insulin signalling pathways regulating glucose transporter translocation and glycolysis, further limiting glucose availability for ATP generation in cardiomyocytes [122, 123]. This creates an energy imbalance, leading to impaired contractile function and promoting adverse myocardial remodelling [51].

In humans, clinical evidence consistently demonstrates impaired glucose handling across laminopathies. In FPLD, insulin resistance affects skeletal muscle, liver, and adipose tissue, leading to ectopic lipid deposition in non-adipose organs and lipotoxicity that interferes with glucose uptake [29, 121, 124, 125]. Even though there are no reports of increased ectopic fat deposition in the heart, this could contribute to the development of heart failure in laminopathy patients. Subcutaneous fat loss lower circulating leptin levels, disrupting lipid and glucose regulatory pathways, further reinforcing systemic insulin resistance [126]. Transcriptomic analyses from FPLD patient myocytes suggest that these observations are the consequence of dysregulated mitochondrial biogenesis and altered muscle protein synthesis pathways [117].

Comparable metabolic disturbances are observed in MAD, where severe insulin resistance frequently progresses to type 2 diabetes [19, 20].

In *LMNA*-related DCM, a study using p.(Glu105Leu) induced pluripotent stem cell (iPSC)-derived cardiomyocytes showed an increase in insulin-independent glucose uptake and reduced oxidative capacity for glycolytic substrates [84].

Furthermore in HGPS, leptin levels have been associated with hyperinsulinemia, insulin resistance and impaired glucose tolerance, contributing to the development of type 2 diabetes [33, 127]. Type 2 diabetes is frequently reported as a clinical manifestation in patients with AWS, and is preceded by insulin resistance, leading to decreased glucose uptake. Additionally, these patients often display β-cell dysfunction, leading to a decreased ability to compensate for insulin resistance [37].

Skeletal muscle involvement in laminopathies also shows a strong glucose-related metabolic component. In LGMD1B, patient muscle biopsies reveal decreased glucose oxidation and downregulation of genes involved in glycolysis and mitochondrial complex I, together with a suggested shift from oxidative glucose utilisation towards increased reliance on incomplete fatty acid $\:\beta\:$-oxidation and increased ketogenesis, possibly contributing to muscle weakness and fatigue observed in these patients [3]. Similarly, EDMD patients-derived myoblasts exhibit reduced glycolytic activity and compromised mitochondrial respiration, which correlates with the severity of hypertriglyceridemia, suggesting an impaired energy production [128, 129].

### Findings from Animal Models

Findings from animal models provide complementary but often contrasting findings on these metabolic disturbances. In the EDMD mouse model (*Lmna*^*∆*32/*∆*32^), hypoglycaemia is a consistent finding, and it is hypothesised that defective SREBP1 signalling contributes to the observed disturbances in glucose metabolism [4]. In HGPS, studies in *Zmpste24*^−/−^ and *Lmna*^G609G/G609G^ mouse models display a different metabolic profile compared to patients, with animals showing hypoglycaemia, hypoinsulinemia, and high glucose tolerance [130, 131]. It is proposed that the observed hypoglycaemia contributes to the development of cardiac arrhythmias in these mouse models [131]. Mechanistic studies in *Lmna*^−/−^ mice indicate that *Lmna* variants can also impair growth hormone signalling through Jak2, Stat5, and Erk pathways, thereby further worsening insulin sensitivity and glucose homeostasis [97, 132]. These differences highlight the limitations of current preclinical systems and translational barriers into the clinical setting.

Beyond these tissue-specific defects, *LMNA* variants directly disturb $\:\beta\:$-cell function and differentiation. SREBP1, which normally regulates adipocyte differentiation, is also essential for pancreatic $\:\beta\:$-cell differentiation and glucose-stimulated insulin secretion. Disrupted SREBP1c activity reduces $\:\beta\:$-cell mass and impairs glucose-stimulated insulin secretion, further aggravating systemic glucose intolerance [97, 133]. Supporting this, studies in mice expressing only lamin C suggest that lamin C plays a protective role in $\:\beta\:$-cell dysfunction by helping maintain $\:\beta\:$-cell mass and insulin secretion as the animals age [134].

The reported species-specific differences in metabolism may arise from intrinsic variations at the cellular, tissue, and system levels. For instance, mice rely more on hepatic glucose disposal while humans depend primarily on skeletal muscle. Both species rely on different glucose transporters, with adult humans primarily relying on GLUT4, which traffics to the plasma membrane via a clathrin isoform that is absent in mice [135]. Mice also exhibit higher mass-specific metabolic rates and weaker homeostatic control of metabolic networks, leading to different aging processes and metabolic disease patterns, compared to humans [136]. Additionally, genetic compensation may influence the disease phenotype observed in animal models. In *Lmna*^−/−^ mice, increased expression of lamin C is observed, which may partly compensate for the complete loss of lamin A, ameliorating the laminopathy phenotype compared to that observed in patients who have different genetic variants in *LMNA* [137].

Taken together, impaired glucose metabolism and systemic insulin resistance appear to be recurring features across diverse laminopathy subtypes, although the clinical expression is heterogeneous. Patient data consistently indicates that *LMNA* variants predispose to insulin resistance, whereas animal models often display different metabolic phenotypes.

## Mitochondrial Abnormalities and Dysfunction in Laminopathies

The heart is known to have a high energy demand and is particularly vulnerable to mitochondrial dysfunction. Impaired mitochondrial oxidative phosphorylation leads to reduced ATP production, which compromises cardiomyocyte ability to contract and contributes to the development and progression of heart failure [138]. Mitochondrial dysfunction can lead to excessive reactive oxygen species (ROS), causing oxidative stress that damages macromolecules, such as DNA, lipids and proteins, and leads to the activation of cell death pathways [139]. Alterations in mitochondrial dynamics, including imbalances in mitochondrial fusion and fission processes, further drive mitochondrial dysfunction and contribute to cardiomyocyte loss [140]. Additionally, impaired mitochondrial Ca^2+^-handling can lead to a disrupted excitation-contraction coupling, leading to arrhythmias and contractile dysfunction (see Fig. 2) [141].

An increasing number of studies implicate mitochondrial abnormalities as a contributor to the pathophysiology of laminopathies [6, 84, 142–145]. Across laminopathies, several mitochondrial abnormalities emerge as recurrent hallmarks, such as dilated and fragmented mitochondria, disorganised cristae, reduced oxidative phosphorylation, and increased ROS production [6, 142]. These features indicated a compromised energy production, increased oxidative stress and impaired mitochondrial quality control. Nonetheless, each laminopathy also exhibit distinct mitochondrial alterations that align with its specific tissue involvement and clinical presentation.

In *LMNA*-related DCM, several studies have provided evidence of mitochondrial abnormalities using animal, patient, and iPSC models [84, 143–145]. Key observations include mitochondrial dilatation in cardiac tissue, and activation of both the Fas and mitochondrial pathway of apoptosis in *Lmna*^E82K^ mice [144]. Similarly, in the *Lmna*^RC/RC^ mouse model mitochondrial dysfunction is observed, characterised by a dilated morphology and a reduced oxidative respiration [146]. Furthermore in HGPS, *Lmna*^*−/−*^ mice reveal a decreased mitochondrial biogenesis, as a result of reduced Sirt1 expression [145]. Additionally, mitochondria have been observed in the nuclei of cardiomyocytes of patients with the *LMNA*^*D192G*^ variant. This remarkable compartmentalisation is likely linked to the underlying structural defects in the nuclear lamina, characteristic of laminopathies [143]. Recent work in *LMNA* p.(Glu105Leu) iPSC-CMs has extended these findings and shown a decreased mitochondrial mass and altered mitochondrial distribution, with mitochondria clumping between sarcomeres in *LMNA*-related DCM [84].

In FPLD, mitochondrial dysfunction contributes to both muscle fatigue and systemic metabolic complications. ^31^P-Magnetic resonance spectroscopy and transcriptomic analyses show reduced oxidative phosphorylation [117, 147], and downregulation of complex I in patient muscle tissue, indicating impaired energy production [3]. Furthermore, fibroblasts carrying the *LMNA* p.Arg439Cys variant show increased oxidative stress sensitivity [148].

In EDMD, mitochondrial dysfunction has been reported at the structural and transcriptional level. Patient myoblasts show a reduced mitochondrial number, leading to a reduced mitochondrial respiration and ATP synthesis [94]. Transcriptomic analyses further showed an upregulation of mitochondrial encoded genes, potentially contributing to increased oxidative stress. In contrast, nuclear-encoded mitochondrial genes were found to be downregulated [94]. This imbalance may increase oxidative stress by the formation of incomplete mitochondrial complexes, causing electron leakage and excess ROS. Given the muscle weakness and progressive contractures in EDMD, reduced ATP synthesis may contribute to the impaired muscle function.

Mitochondrial dysfunction is one of the hallmarks of aging [149], aligning with the mitochondrial observations in HGPS. In a HGPS mouse model (*Lmna*^G609G/G609G^), multiple alterations in the mitochondrial metabolism have been observed, including a downregulation of proteins involved in oxidative phosphorylation leading to a decreased ATP synthesis [33, 150]. This is supported by the observed decrease in oxygen consumption rate, indicating an overall downregulation of mitochondrial respiratory function in HGPS patient fibroblasts [151]. Moreover, the mitochondrial biogenesis is impaired in HGPS, leading to a reduced mitochondrial mass which may further contribute to the compromised ATP synthesis in HGPS patient fibroblasts [152]. Furthermore, the mitochondria appear dilated and fragmented, along with decreased mitochondrial movement [33, 152]. In addition to the decreased mitochondrial function, an increased ROS generation has been associated with cellular senescence and apoptosis [33, 153, 154]. Interestingly, mitochondrial dysfunction has also been observed in the early stages of HGPS mouse models (*Lmna*^*G609G/G609G*^, *Zmpste*^*−/−*^) and patient fibroblasts [150, 152, 155–159]. Additionally, perinuclear mislocalisation of mitochondria has been observed in HGPS fibroblasts [142]. However, it is important to mention that the HGPS phenotype is only fully recapitulated in homozygous mouse models, not expressing wildtype lamin A. Whereas in humans, HGPS patients carry a heterozygous variant. This difference necessitates cautious interpretation of mouse model findings, as their relevance to the human condition is limited [160, 161].

Notably, studies in the HGPS mouse model (*Lmna*^*G609G/G609G*^) yielded conflicting results regarding energy expenditure. Some reports describe increased energy expenditure, reflected by higher respiration rates (VO2 and VCO2) compared to wildtype mice [162]. In contrast, others observed decreased energy expenditure, measured as reduced heat consumption in *Lmna*^*G609G/G609G*^ mice compared to controls [163]. These observed differences may be partly attributed to the differences in methodologies used to assess energy expenditure.

## Therapeutic Approaches Targeting Metabolism

### Current Therapies in *LMNA*-related Dilated Cardiomyopathy

In the context of heart failure management in laminopathy patients, specific recommendations have been developed to address the clinical challenges associated with laminopathies [164]. For patients with *LMNA*-related DCM, conventional heart failure therapies are typically employed but with special considerations to the disease’s unique pathophysiology and risks, which can affect drug responses [165, 166]. General heart failure therapy including beta-blockers, inhibition of the RAAS pathway (angiotensin converting enzyme (ACE) inhibitors, angiotensin receptor blockers (ARB) or angiotensin receptor neprilysin inhibitor (ARNI), mineralocorticoid receptor antagonist (MRA), and sodium-glucose cotransporter protein 2 (SGLT2) inhibitors are recommended as a first-line treatment to reduce cardiac remodelling and improve left ventricular functions, and to reduce arrhythmic events, which are common in laminopathies [48, 164, 167]. Furthermore, early consideration of an implantable cardioverter-defibrillator (ICD) placement is recommended for primary prevention of sudden cardiac death, even in patients with a (low-)normal systolic function, due to the high risk of arrhythmic events associated with *LMNA* variants [48, 168]. Decision-making for ICD implantation is guided using a specific *LMNA* risk calculator, which incorporates multiple clinical risk factors of personalised risk stratification [164, 169]. However, in cases where heart failure progresses despite medical and device management, heart transplantation is required. Nearly 20% of laminopathy patients require heart transplantation during follow-up [167, 170]. A *LMNA*-specific therapy aiming to inhibit p38 (ARRY-371797) was promising but did not show a beneficial effect yet in recent clinical trials [171, 172].

### Metabolic-Targeted Strategies in Laminopathies

While conventional therapies aim to aid cardiac function, emerging studies suggest that modulating specific metabolic pathways may offer additional benefits in managing *LMNA*-related DCM. For example, enzymatic therapies, such as modulating glucose-derived serine and glycine biosynthesis has been proposed as a therapeutic approach for *LMNA*-related DCM [173]. Recent studies in iPSC-CM models carrying various DCM genetic variants, including in *LMNA*, and a DCM mouse model suggest that alterations in these metabolic pathways contribute to the pathogenesis of DCM [173–175]. By targeting enzymes involved in serine and glycine synthesis, such as phosphoglycerate dehydrogenase (PHGDH) and serine hydroxymethyltransferase 2 (SHMT2), an increase in the mitochondrial respiration and ATP synthesis have been observed in *LMNA*-related DCM iPSC-derived cardiomyocytes, resulting in an improved contractile function [173–176].

Beyond these enzymatic interventions, several metabolic strategies are being explored across the spectrum of laminopathies, including pharmacological agents, dietary interventions, mitochondrial-targeted therapies, gene therapy and protein modulations. Pharmacological agents targeting metabolic pathways demonstrate promising therapeutic potential for laminopathies in a prospective case series reporting 5 patients. PPARγ agonists, such as thiazolidinediones, exert modest improvements in glucose metabolism, presumably by promoting adipocyte differentiation, which could be beneficial in lipodystrophy-related laminopathies, as shown in study using a adipose cell lineage tracing mouse model [177, 178]. PPARγ agonists work by activating the PPARγ nuclear receptor, a key transcription factor that regulates genes involved in adipogenesis, lipid uptake, and glucose homeostasis. Thus, activation of PPARγ enhances the formation of functional adipocytes, increases insulin sensitivity in peripheral tissues, and promotes the storage of lipids in adipose tissue rather than in ectopic fat deposits [179, 180].

Similarly, metformin has been shown to enhance insulin sensitivity and reduce oxidative stress in FPLD and EDMD [17, 181, 182]. Metformin activates AMPK, leading to the upregulation of downstream targets such as SIRT1 and SIRT3, which in turn enhance the activity of PGC-1$\:\alpha\:$,the master regulator of mitochondrial biogenesis [183–185]. This results in increased mitochondrial DNA content improved oxidative capacity, and reduced oxidative stress in various cell models, including rat cardiomyocytes and human umbilical vein endothelial cells (HUVEC) [183–185]. Multiple preclinical studies in diabetic cell and mouse models show that metformin preserves mitochondrial membrane potential, promotes mitochondrial dynamics, and delays cellular senescence [186–188]. For instance, metformin treatment in a Duchenne muscular dystrophy (mdx) mouse model has been shown to improve muscle strength, enhance sarcolemma integrity, increase neuromuscular transmission, and upregulate AMPK phosphorylation and dystrophin-associated complex proteins [181]. However, a clinical trial combining metformin with the nitric oxide precursor L-citrulline in Duchenne muscular dystrophy patients has shown only modest benefits, with significant motor function improvements observed only in the stable subgroups [182, 189]. Moreover, while most studies underscore the positive effects of PPAR$\:\gamma\:$ agonists on mitochondria, limited animal models suggest that excessive mitochondrial activation may not always translate to improved viability or may induce compensatory stress responses [190–192].

Other insulin sensitisers, including incretin mimetics, are also being examined for their use in laminopathy treatment. Incretin mimetics, such as Glucagon-Like Peptide-1 (GLP-1) receptor agonists, have shown potential to improve glucose regulation and lipid profiles in *LMNA*-related lipodystrophy syndromes, particularly by enhancing insulin sensitivity and addressing metabolic dysregulation. This evidence primarily derives from a small retrospective study in FPLD patients [193, 194]. GLP-1 receptor agonists work primarily by mimicking the action of endogenous glucagon-like peptide-1, binding to the GLP-1 receptor on pancreatic $\:\beta\:$-cells to stimulate glucose-dependent insulin secretion, while simultaneously inhibiting glucagon release from $\:\alpha\:$-cells. This lowers blood glucose levels, especially after meals [195, 196]. In addition, statins inhibit HMG-CoA reductase, lowering the levels of low-density lipoprotein (LDL) cholesterol. They have been shown to improve lipid profiles and reduce cardiovascular risk in FPLD and HGPS patients in phase 2 clinical trials [17, 197].

Currently, dietary interventions are used complementary to pharmacological treatments in laminopathies. For instance, in patients with lipodystrophy with severe hypertriglyceridemia, caloric restriction may be used to lower lipid serum levels and mitigate the risk of metabolic complications associated with excess fat accumulation. While caloric restriction reduces overall energy intake, it can help balance abnormal lipid metabolism in these patients [198–201]. In contrast, patients with HGPS and EDMD, who often experience increased energy demands due to muscle wasting or other metabolic challenges, are recommended to follow a high-calorie diet [163, 202].

Interestingly, preclinical studies have shown the potential benefits of high-fat diets in specific laminopathy models. For example, in a HGPS mouse model (*Lmna*^*G609G/G609G*^*)*, a high-fat diet composed of 60% calories from fat resulted in a greater increase in lifespan than any other therapeutic intervention tested to date [163]. However, it is important to note that high-fat diets could carry other risks, such as triggering arrhythmias or atherosclerosis. For instance, altered myocardial fatty acid handling, can lead to excessive lipid uptake and lipotoxicity, impairing calcium homeostasis and increase the susceptibility to electrical conduction disturbances [203, 204]. Additionally, high-fat diets may accelerate vascular pathology by elevating circulating LDL cholesterol and promoting endothelial dysfunction, which can be drivers of atherosclerotic plaque formation [205, 206]. Given that HGPS patients already have an increased risk for atherosclerosis due to vascular smooth muscle cell depletion and arterial stiffening, the long-term effects of high-fat diets on vascular health should be investigated [207, 208]. In addition, supplementation strategies have shown promise in preclinical models. For instance, studies using a transgenic *Danio rerio* model of muscular dystrophy have showed the benefits of *L*-carnitine and creatine supplementation, which enhances lipid metabolism and ATP synthesis in muscle cells [209].

Given the role of mitochondrial dysfunction in laminopathies, therapies specifically targeting mitochondria are of interest. Mitochondrial-targeted antioxidant therapies, such as lysophosphatidic acid (LPA) have been shown to reduce oxidative stress and improve mitochondrial function in HGPS patient fibroblasts and a zebrafish model [210, 211]. Additionally, strategies that enhance mitochondrial biogenesis have been investigated in the context of laminopathy treatment. For instance, resveratrol and sirtuin activators, which activate PGC-1α may offer benefit by increasing mitochondrial biogenesis and improving energy production in a transgenic HGPS mouse model [212, 213].

In summary, while conventional therapies remain the current management approach in *LMNA*-related DCM, emerging metabolism-targeting therapies may hold the potential to advance treatment options and improve long-term outcomes across the broader spectrum of laminopathies (Table 2).

**Table 2:** Metabolic-Targeted therapeutic approaches in Laminopathies. This table presents an overview of current and emerging therapeutic strategies targeting metabolic pathways in *LMNA*-related dilated cardiomyopathy (DCM) and other laminopathies. Evidence includes findings from preclinical models (iPSC-derived cardiomyocytes (CMs), mouse models, and zebrafish models) and clinical cohorts, highlighting how metabolic targeted interventions improve mitochondrial function, energy metabolism, and cardiac outcomes

Target	Therapy	Evidence	Model
Targeted Experimental Therapies			
p38 MAPK pathway	ARRY-371,797 (p38 inhibitor)	Promising preclinically, no benefit in clinical trials	*Lmna*^H222P/H222P^ mouse model, phase III clinical trial^171,172^
**Enzymatic Therapies**			
Serine/glycine biosynthesis	PHGDH/SHMT2 modulation	Improves mitochondrial respiration, ATP synthesis, contractile function in iPSC-CMs	*LMNA* variant iPSC-CMs, DCM mouse model [173–176]
**Pharmacological Metabolic Modulators**			
Adipocyte differentiation/metabolism	PPAR$\:\gamma\:$ agonists (thiazolidinediones)	Modest improvements in glucose metabolism, promotes adipogenesis	Adipose cell lineage tracing mouse model, prospective case series (5 FPLD patients) [177, 178]
Insulin sensitivity/mitochondria	Metformin	Enhances insulin sensitivity, AMPK/SIRT1/SIRT3/PGC-1$\:\alpha\:$ activation, improves mitochondrial function	Cell culture (rat cardiomyocytes, HUVECs), mdx mouse model, clinical pilot [182–189]
Glucose/lipid regulation	GLP-1 receptor agonists	Improves glucose/lipid profiles in lipodystrophy, enhances insulin sensitivity	Retrospective FPLD patient study [193, 194]
Lipid metabolism	Statins (HMG-CoA reductase inhibitors)	Improves lipid profiles, reduces cardiovascular risk in FPLD/HGPS	Phase 2 clinical trial (FPLD, HGPS patients) [17, 197]
**Dietary and Supplementation Strategies**			
Caloric intake	Caloric restriction	Lowers serum lipids, mitigates metabolic complications in lipodystrophy	Clinical (lipodystrophy patients) [198–201]
Energy balance	High-calorie diet	Recommended in HGPS/EDMD with increased energy demands	Clinical recommendations (observational data) [163, 202]
High-fat diet	60% fat diet	Extends lifespan in HGPS mouse model, but risk of arrhythmias/atherosclerosis	HGPS mouse model (*Lmna*^G609G/G609G^) [163]
Lipid metabolism/ATP synthesis	L-carnitine, creatine supplementation	Enhances lipid metabolism, ATP synthesis in muscle	Transgenic *Danio rerio* muscular dystrophy model [209]
**Mitochondrial-Targeted Therapies**			
Mitochondrial oxidative stress	Mitochondrial targeted antioxidants (LPA)	Reduces oxidative stress, improves mitochondrial function	HGPS fibroblasts and *Lpa3*^−/−^ zebrafish model [210, 211]
Mitochondrial biogenesis	Resveratrol, sirtuin activators	Activates PGC-1$\:\alpha\:$, increases mitochondrial biogenesis, improves energy production	HGPS transgenic mouse model [212, 213]

## Conclusion

Metabolic dysregulation is a common feature across various laminopathies, manifesting not only in the late stages but also early in the disease progression. Several studies have reported alterations in lipid metabolism, glucose homeostasis and mitochondrial function in both preclinical laminopathy models but also in patients. The spectrum of metabolic alterations observed across various laminopathy phenotypes suggests that they should not be viewed entirely as distinct clinical entities but as a continuous spectrum of disorders with overlapping mechanisms. Nevertheless, comprehensive metabolomic studies across different laminopathy subtypes have not been performed yet.

Recent advancements have been made in understanding the underlying molecular mechanisms in laminopathies, bringing forward potential new therapeutic targets. Several preclinical studies targeting the metabolic alterations in laminopathies have shown beneficial results. However, the translation to clinical practice remains limited and only a few patient studies have been completed. There is a growing need for innovative therapeutic approaches and targeting metabolic pathways may potentially improve outcomes for laminopathy patients.

## Data Availability

No datasets were generated or analysed during the current study.
